# Development and validation of the relational behavior interactions scale for couples

**DOI:** 10.1038/s41598-024-58901-2

**Published:** 2024-04-06

**Authors:** Tal Harel, Meni Koslowsky

**Affiliations:** 1 Department of Psychology, Bar Ilan University, 5290002 Ramat Gan, Israel; 2 Department of Psychology, Ariel University, Ariel, Israel

**Keywords:** Behavior interactions scale, Gender similarities, Measurement, Psychometrics, Human behaviour, Health occupations

## Abstract

In this research, we developed and validated a measure of couple-based reported behavior interactions (RBI). Specifically, Study 1 was designed to describe the development of the scale and to examine its reliability; Study 2 (N = 222), was designed to examine factors that could differentiate men and women. Additionally, we tested if women's behaviors could predict their partner's behavior. Results from the exploratory factor analysis (EFA) revealed a three-factor structure for couples' RBI which were labelled: Social Companionship and Affective Behavior Interactions (SAI) (Factor 1), Fulfilling Obligations and Duties of the Partner (FOD) (Factor 2) and Openness in the Relationship (OR) (Factor 3). In linear regression analyses, there was a significant difference between men and women in the second factor, which represents behaviors associated with fulfilling the responsibilities of a partner. On the other hand, neither the SAI factor nor the OR factor showed any distinct gender differences. The SPSS PROCESS analysis revealed that women's Social Companionship and Affective Behavior Interactions (Factor 1), and Openness in the Relationship (Factor 3) significantly predicted their male partner's behaviors. The relationship duration significantly moderated the association between women's and men's behaviors for both factors. Results are discussed in light of the need for a broader understanding of romantic behavioral interactions.

## Introduction

The literature on romantic relationships has grown considerably during the ensuing years. Indeed, a December 2023 APA PsycNet search yielded 2191 references appearing since 2013 that mention either “romantic behaviors” or “romantic interaction”. Romantic relationship behaviors thus continue to attract scholarly attention. In forming and maintaining relationships, individuals develop expectations about how they ought to be and ought to behave^1,2^. Interactions that have been widely studied in married couples include positive (e.g., acceptance, recognition, compromise, and support) and negative (e.g., blame, criticism, coercion, and anger) behaviors^3,4^. Studies have reported several patterns of behaviors that are predictive of relationship satisfaction and dissolution^5,6^. Others have found associations between commitment and different sorts of behaviors including maintenance behaviors, accommodation, forgiveness, and cognitive interdependence^7,8^. Common behaviors found in friendships and romantic relationships include emotional closeness, intimacy, mutual disclosure, loyalty, respect, and unique behavior for each other^9^.

There is a lack of research on gender differences in behavioral interactions between long-term romantic partners, so the present study sought to clarify these differences. A gender perspective is crucial for understanding couples' behaviors as their respective roles shape how both men and women function in society, including their roles in the family and at work^10^. Social role theory suggests that people develop certain stereotypes or beliefs about their gender roles by observing how men and women behave and assume that each partner possesses specific characteristics. For instance, in industrialized societies, women often take on nurturing roles both at work and at home. This leads to a belief that women are caring and communal individuals. Consequently, based on social role theory, men and women are inclined to display distinctive behaviors in their relationships^10^.

Social role theory also incorporates the idea that differences in social behavior between the sexes, such as how they respond to various situations, stem from how they perceive their specific societal roles^11,12^. Specifically, it suggests that men are typically taught to be independent and to confront challenging situations, like conflicts in relationships, directly, often with aggression or coercion^13^. On the other hand, women, who are usually taught to adhere to traditional gender roles, are encouraged to be communal, expressive, and dependent^12,14,15^.

According to more traditional gender role attitudes, men and women are expected to perform a variety of behaviors in society according to their distinct gender roles (e.g., men should be employed in the labor force as breadwinners and women should be employed as nurturers). The egalitarian gender role attitude holds that men and women can occupy the same role in any given setting and encourages deviation from traditional gender roles^15^.

To date, several papers have contributed to defining and measuring relationship behaviors^16^. For example, Canary and Stafford^17^ developed a five-category-typology of relational maintenance behaviors that are actions and activities used to sustain desired relational qualities. Their categories including positivity, openness, assurances, and sharing tasks were the basis of their scale^17^. In a later work, Fuhrman et al.^18^ identified three sets of behaviors that we expect from all romantic relationships namely emotional closeness, social companionship, and relationship positivity replicate the most common of those identified in previous research^19,20^. Weigel et al.^21^ identified common behaviors people use to indicate their level of commitment to their partners.

Surprisingly, there is no current, comprehensive, psychometrically sound measure of romantic behavior interactions. Current measures of romantic behaviors are creditable but incomplete. While the present measures of romantic behaviors are commendable, they are not exhaustive. Some, like those developed by Canary^17^, Fuhrman et al.^18^, and Weigel et al.^21^, only assess the maintenance component or identify positive behaviors related to the emotional closeness/affection aspects of a marriage. Other relationship scales, such as the Romantic Partner Conflict Scale (RPCS)^22^, evaluate conflict behavioral strategies that may either promote or harm unmarried romantic couples. The Romantic Jealousy-Induction Behaviors^23^ assess specific behaviors of romantic jealousy. The Affectionate Communication Index^24^ measures affectionate communication within a relationship. As such, a measure that captures various romantic behavior interactions could be beneficial in understanding couples’ romantic experiences.

The necessity of constructing a new scale arises from the gaps in previous studies, which did not adequately consider various behaviors that significantly relate to relationship satisfaction and commitment in a relationship^25^. These behaviors include fulfilling the obligations and duties of the partner, such as accepting the partner’s requests and needs, adhering to joint agreements, and fulfilling household tasks. Another crucial behavior is openness in a relationship, which involves discussing personal needs, concerns, feelings, and emotions with one's partner. Moreover, previous measures of romantic behaviors have not differentiated between the behaviors exhibited by women and men, nor have they focused on long-term relationships. Our new scale addresses these gaps by considering these factors and may help in providing a more comprehensive understanding of relationship dynamics. Our scale may be a valuable addition to the field as it may help to provide a better understanding of the factors that contribute to relationship satisfaction and commitment.

The present study is designed to develop the Relational Behavior Interactions (RBI) scale which will enable a better means for investigating different aspects of romantic behavior interactions in couples within a long-term romantic relationship. These interactions encompass affective behaviors such as openly conveying warmth, offering compliments, and displaying love. We also consider behaviors associated with couples fulfilling obligations and duties. This includes accepting partner requests and needs, as well as carrying out tasks and responsibilities in the domestic sphere. Furthermore, the study delves into behaviors associated with openness in the relationship including the sharing of emotions and feelings with one's partner while engaging in open conversations on a range of subjects. Our primary objective here is to assess and characterize the psychometric properties of the RBI scale, as well as to determine whether these behaviors can distinguish between men and women in the context of romantic relationships. In addition, we plan to investigate the significance of gender differences in scale factors.

## Literature review

### Reported behavior interactions

Behavioral interactions are characterized as the actions that individuals genuinely exhibit with their partners which are supposed to assist individuals in defining and achieving their relationship objectives^26,27^. Several studies have underscored the significance of romantic behaviors in relationships. Investigations in the area of family research have recognized the pivotal role of interaction patterns within couples and how these couples perceive those interactions in determining the ultimate success or failure of relationships^28,29^. For instance, the vulnerability-stress-adaptation model by Karney and Bradbury^30^, and the romantic relationship development model by Bryant and Conger^31^, both highlight the importance of couple interactions, particularly hostile ones. Conger et al.^32^ model showed a correlation between a young adult’s hostility towards a romantic partner and low relationship quality.

One challenge that family researchers face in studying behavioral interaction patterns is that they manifest themselves in different ways, often a function of who is reporting the behavior. Hence, when a single individual answers all the questions in a survey, the responses may be skewed by that individual’s unique focus. Gathering information about romantic relationships from a single respondent may lead to systematic measurement errors, specifically, method variance bias^33^. However, most studies probing this issue have concentrated on young adults’ dating relationships or newlywed couples, with very few studies testing long-term relationships^34–38^. By using previous research in the field as a theoretical basis, the current study investigated differences in romantic behaviors as reported by couples in long-term romantic relationships. Moreover, the study examined gender differences in reported behavioral interactions.

### Gender similarities in couple's behavior interactions

Previous investigations have shown there are some similarities between women and men in several relational aspects including thinking, feeling, and behaving. Regarding emotionality, men experience emotions similar to women and differ in how they are expressed^39^. Some studies have shown that women express more positive and negative emotions than men do^40,41^, while others found no differences across partners in either positive or negative emotional expressivity^42,43^. Furthermore, studies have shown that women are more expressive than men during relationship problem discussions and are more prone to verbalize their negative emotions^44^. Furthermore, Gottman et al.^28^ reported that women in romantic relationships are generally more open in their expressions compared to men, as the latter group tends to retreat from interaction when they experience physiological arousal^28^. Studies that examined maintenance behaviors have found links with biological sex and gender roles^45,46^. For example, women performed more maintenance behaviors than men, but this finding was consistent for only two of the behavioral factors: sharing tasks and openness^45,47^. Other researchers argue that there was a weak link between gender and these behaviors^46^.

As the literature in this area contains many inconsistencies regarding men's and women's behaviors in romantic relationships^48,49^, a major goal here is to determine whether the current scale could be used to distinguish between behaviors within partners. The present study also supplements previous findings in this area by examining additional types of couples' behaviors as a function of gender.

### Relationship duration

Another important variable to be considered here is relationship duration, which is crucial for understating how relational processes change across development. Relationship duration is the relative time investment that partners have made to their relationships^16^. The link between relationship duration and a couple's behavioral interactions remains unclear. Some studies have shown that the use of openness, positivity, and assurance behaviors decreases with relational length^46,50^, while the use of sharing tasks and social network behaviors were unrelated to relationship duration^51,52^. As such, the present research also examines whether relationship duration plays a moderating role in the relationship between women's and men's interactions (i.e., social companionship and affective behavior, fulfilling the partner's obligations and duties, and being open with the partner).

### Study goals

To date, most questionnaires for assessing relationship behaviors have been designed to measure certain types of behaviors (e.g., maintenance behavior, coping behavior, and intimacy)^18,53^. We suggest, however, additional factors of behavior interactions, such as sharing values, coping with problems, expressing thoughts and feelings. Accordingly, we developed a measure of couples-based RBI, that assessed a variety of behavior interactions reported by both partners in long-term romantic relationships. In this study, the partner's gender and relationship duration are included in the scale analysis.

Firstly, the construction and validation of the RBI scale are presented. The Pilot Study (Study 1) was designed to develop the RBI scale and determine its reliability; The purpose of Study 2 was to assess and characterize the scale's psychometric properties, as well as to examine whether there are significant differences in scale factors between men's and women's behaviors. Finally, the study tests whether women's responses could predict their partner's behaviors and ascertain the extent to which relationship duration moderates these associations.

## Method

### Study 1

#### Participants

The Pilot Study included 30 Israelis aged 20 to 55, half of whom are female and half male, who have been in a romantic relationship for at least a month (M = 86.83, SD = 100.17). The participants were volunteers who willingly agreed to take part in the study. All participants signed informed consent prior to participation. The exclusion criteria were: (a) unemployed (b) involved in a romantic relationship for less than one month. All the experimental protocols of the current study were approved by Bar-Ilan University IRB ethics committee. In addition, all methods were carried out in accordance with relevant guidelines and regulations.

#### Materials and procedure

The questionnaire included items assessing couples-based RIB that occurred in the current relationships. To date, behaviors that are based on Canary et al.^17^ and Fuhrman et al.^18^ dimensions, were less focused on each of the couple behaviors and gender differences. We followed Hinkin^54^ and Boateng et al.^55^ approaches to develop the scale. Initially, a pool of 27 potential items was generated to measure couples' behaviors in their current relationship. Items were based on a previous literature review and later refined to identify ambiguous and unclear wording. This set of items was then further refined by presenting our definitions of a RBI scale to a pool of topic experts who decided whether each of the items was relevant. That process of matching items with definitions occurred several times until all raters agreed that our final set of items aligned with the construct in question.

To determine the psychometric properties of the scale a Pilot Study was conducted. Participants were required to read 27 items in this version of the scale and respond on a 5-point scale ranging from *"Strongly Disagree*" to "*Strongly Agree*" as to how well each of the items describes their behavior in the current relationship *(Cronbach α* = 0.81). Scale reliability test after removing two items yielded a *Cronbach α* = 0.92 leaving the final scale with 25 items.

## Study 2

Study 2 was designed to examine the RBI factorial structure, convergent validity, and whether the scale could differentiate between men's and women's partners' behaviors. Additionally, we examined whether women's responses could predict their partner's behaviors. Furthermore, relationship duration was tested as a moderator.

### Method

#### Participants

For Study 2, the revised questionnaire was sent to a sample of 111 Israeli heterosexual couples (N = 222), who have been in a romantic relationship for over a year. The mean age of the respondents was 34.62 years (SD = 8.50) and the average time in the relationship was 9 years (M = 9.96, SD = 7.01). The vast majority were college-educated (95.5%) while 4.5% were secondary-educated.

#### Materials and procedure

Based on comments made by the respondents in the Pilot Study, the participants were asked to indicate how well the items describe behaviors that occurred in their relationship during the past week, for example, *"I hugged my partner", "I gave my partner some space so he/she could focus on things that are important to him/her",*

*"I mentioned to my partner how unique our relationship is, unlike other people's relationships".* The respondents were asked to rate the items on a 5-point Likert scale ranging from “Not at all” (1) to “Very much.” (5) The scale yielded a reliability coefficient of *Cronbach's α* = *0.90* for men and α = 0.89 for their women's partners. All the experimental protocols of the current study were approved by the university IRB ethics committee. In addition, all methods were carried out in accordance with relevant guidelines and regulations. We collected data through snowball sampling techniques such as sending the link through a list of e-mail contacts, and these contacts were then asked to forward the e-mail to their contacts. All participants signed informed consent prior to participation. The participants were allowed to drop out of the study at any time. The online data were collected using *Google forms* software, which ensures anonymity and confidentiality of respondents. Couples who agreed to participate in the study completed the questionnaire independently of each other. The approximate time of involvement in the study was 15 min. The data were recorded in an anonymous fashion.

#### Analytical strategy

To explore the factor structure of the scale developed to measure couples' behavioral interactions, exploratory factor analysis (EFA) with VARIMAX rotation was used to determine the number of significant components and item factor loadings (see Table 1). In order to assess the convergent and discriminant validity of the core variables, we conducted a confirmatory factor analysis (CFA) using structural equation modeling (SEM). Two models were included with separate behavioral interactions latent variables for men and women. Additionally, we conducted Pearson correlations between the men's and women's factors in order to assess whether the scale factors could differentiate between men and women partners. As a final step, we used the SPSS PROCESS procedure (model 1) and 5000 bootstrap samples to examine whether women's behaviors predicted their partners' behaviors and how relationship duration moderated these associations.

**Table 1:** Pattern matrix resulting from exploratory factor analysis.

Scale item	Social companionship and affective behavior interactions (SAI) (Factor 1)	Fulfilling obligations and duties of the partner (FOD) (Factor 2)	Openness in the relationship (OR) (Factor 3)
Item 3 I hugged my partner	0.66		
Item 8 I praised my partner for good things he/she did	0.53		
Item 9 I told my partner that I love him/her and expressed my feelings for him/her	0.75		
Item 14 I showed my partner how much I love him/her	0.69		
Item 21 I spent time together with my partner	0.61		
Item 22 I made my partner feel good	0.67		
Item 25 I complimented my partner	0.67		
Item 1 I accepted my partner's requests and needs		0.55	
Item 2 I gave my partner some "space" so he/she could focus on things that are important to him/her		0.49	
Item 4 I followed the joint agreements I have with my partner		0.51	
Item 13 I fulfilled my tasks and duties at home		0.73	
Item 18 Even when we had arguments and fights last week, I have taken actions to preserve our relationship		0.45	
Item 19 In my daily actions, I considered my partner		0.71	
Item 23 I helped my partner last week when he/she asked for help		0.57	
Item 10 I had the chance to discuss shared goals, plans, and ambitions with my partner			0.58
Item 11 I mentioned to my partner how unique our relationship is, unlike other people's relationships			0.42
Item 12 I got help from my partner			0.49
Item 15 I talked to my partner about my needs and what bothers me in our relationship			0.69
Item 16 I shared my feelings and emotions with my partner			0.70
Item 20 My partner and I talked openly about a variety of topics			0.46

*RBI* reported behavior interactions.

### Ethical approval

This study was conducted in compliance with ethical standards of the University IRB ethics committee. The data were recorded in an anonymous fashion.

### Informed consent

All participants signed informed consent prior to participation.

## Results

**Figure 1:**
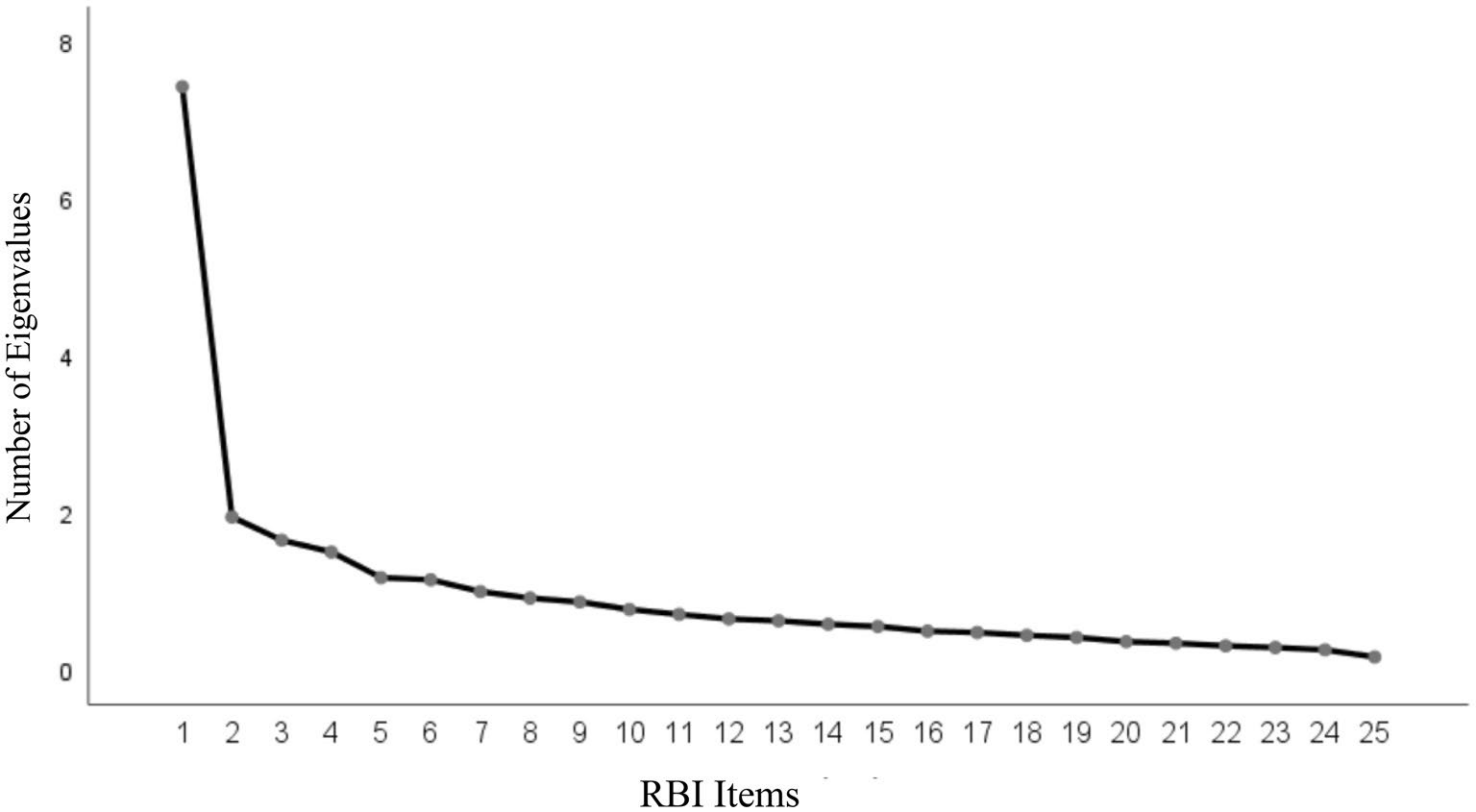
The results of the exploratory factor analysis (EFA). Using a scree plot of the eigenvalues, significant components were identified in accordance with eigenvalues > 1.0. This VARIMAX rotation revealed three significant components that explained 44.08% of the variance.

As a first step, using SPSS 25 an exploratory factor analysis (EFA) was conducted. As shown in Fig. 1, the number of significant components was determined by a scree plot of eigenvalues greater than unity, rather than the traditional value of 1.0^56^. VARIMAX rotation revealed three significant components that explained 44.08% of the variance. Table 1 presents the measurement items with their respective factor loadings. Using a criterion of a loading greater than 0.40^57,58^, we found that Factor 1 reflects social companionship and affective behavior interactions (e.g., showing affection for one another) This factor yielded a reliability coefficient of *Cronbach's α* = *0.84.* Factor 2 reflects fulfilling obligations and duties of the partner (e.g., giving aid and assistance). This second factor yielded a reliability coefficient of *Cronbach's α* = *0.77.* Finally, Factor 3 reflecting openness in the relationship (e.g., sharing thoughts/feelings) yielded a reliability coefficient of *Cronbach's α* = *0.73.*

Additional second-order Exploratory Factor Analysis (EFA) was conducted, excluding two items (12 and 20) and focusing on a three-factor model of 18 items. The analysis showed that factor loadings were improved for each relevant item, aligning with parsimony, leading to more robust results. Therefore, we had a distribution of 7-7-4 items across three factors. The Pattern Matrix resulting from this Three-dimensional Exploratory Factor Analysis with a short version of the 18 items is shown in Table 2.

**Table 2:** Three-dimensional Exploratory Factor Analysis with a short version of the 18-item.

Scale item	Social companionship and affective behavior interactions (SAI) (Factor 1)	Fulfilling obligations and duties of the partner (FOD) (Factor 2)	Openness in the relationship (OR) (Factor 3)
Item 3 I hugged my partner	0.73		
Item 8 I praised my partner for good things he/she did	0.54		
Item 9 I told my partner that I love him/her and expressed my feelings for him/her	0.79		
Item 14 I showed my partner how much I love him/her	0.69		
Item 21 I spent time together with my partner	0.52		
Item 22 I made my partner feel good	0.62		
Item 25 I complimented my partner	0.64		
Item 1 I accepted my partner's requests and needs		0.63	
Item 2 I gave my partner some "space" so he/she could focus on things that are important to him/her		0.54	
Item 4 I followed the joint agreements I have with my partner		0.59	
Item 13 I fulfilled my tasks and duties at home		0.70	
Item 18 Even when we had arguments and fights last week, I have taken actions to preserve our relationship		0.47	
Item 19 In my daily actions, I considered my partner		0.75	
Item 23 I helped my partner last week when he/she asked for help		0.58	
Item 10 I had the chance to discuss shared goals, plans, and ambitions with my partner			0.54
Item 11 I mentioned to my partner how unique our relationship is, unlike other people's relationships			0.63
Item 15 I talked to my partner about my needs and what bothers me in our relationship			0.80
Item 16 I shared my feelings and emotions with my partner			0.72

*RBI* reported behavior interactions.

In addition, a confirmatory factor analysis (CFA) using structural equation modeling (SEM) to assess the convergent and discriminant validity of the core variables was conducted. We included two models with three latent variables (a) Social companionship and affective behavior interactions; SAI (b) Fulfilling obligations and duties of the partner; FOD and (c) Openness in the relationship; OR for men and women separately. The findings for women yielded high factor loadings, i.e., greater than 0.40^59^ for each item with their behavioral factors (see SI Fig. 1 in the supplementary materials for a detailed analysis). The women's model fits the data well: (χ^2^(164) = 235.319, p < 0.001, CFI = 0.91, TLI = 0.90, and RMSEA = 0.06)^60^. Additionally, the results for men indicated high factor loadings (greater than 0.40) for each item with the behavioral factors (see SI Fig. 2 in the supplementary materials for a detailed analysis). The men's model also fit the data well: χ^2^(158) = 219.776, p < 0.001, CFI = 0.92, TLI = 0.90, and RMSEA = 0.06.

We conducted a series of Pearson correlations between the men's and women's factors to examine if the factors were consistent across genders. Factor 1 and factor 3 are relatively matched across gender, the correlation between men and their women partners on factor 1 was (*r* = 0.48*, p* < 0.001) and on factor 3 was (*r* = 0.30*, p* < 0.01). On factor 2, there was no significant correlations between the gender (*r* = 0.05*, p* = 0.56).

To investigate gender differences in the three relational behavior factors, a simple linear regression was employed to determine if gender significantly predicted the levels of each behavioral factor. The findings indicated a significant difference between men and women in the second factor, which represents behaviors associated with fulfilling obligations and duties of the partner. The overall regression of Factor 2 was statistically significant (R^2^ = 0.04, F(1, 109) = 4.73, p < 0.05). It was found that gender significantly predicted Fulfilling Obligations and Duties (FOD) (β = 0.20, p < 0.05). Conversely, the First factor which includes behaviors associated with Social Companionship and Affective Behavior Interactions (SAI), as well as the Third factor, which involves behaviors of Openness in the Relationship (OR), did not exhibit any distinct gender differences.

**Figure 2:**
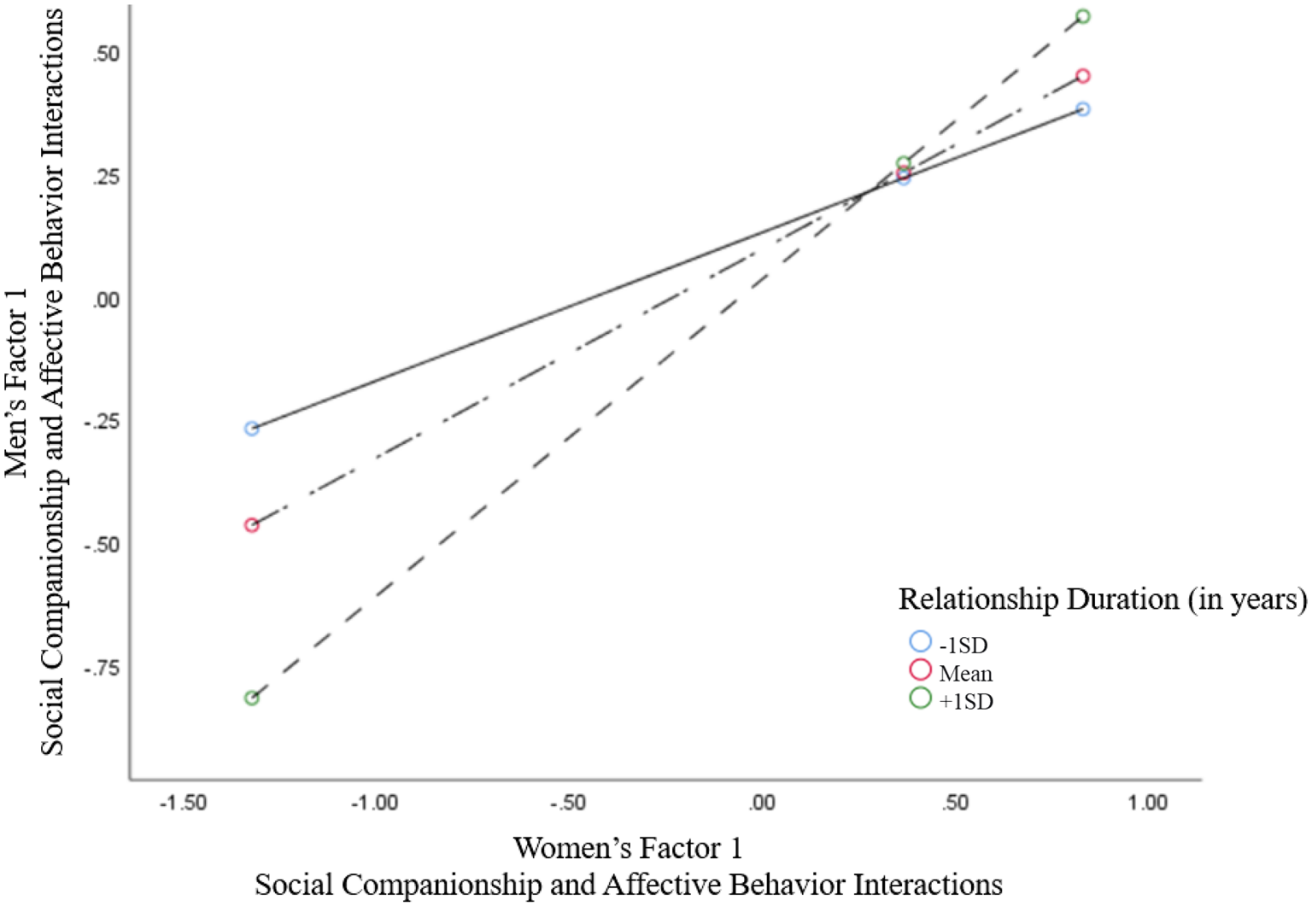
The results of the SPSS PROCESS procedure are presented. The interaction between the women's factor 1 and the relationship duration was significant in predicting the behavior of men. Conditional effects (simple slopes) of women's factor 1 (Social Companionship and Affective Behaviors) on men's factor 1 behaviors at various values of relationship duration (plus (+ 1 SD) and minus (-1 SD) one standard deviation from the mean).

To examine if women's Factors 1 and 3 could be associated with men's behavior interactions and whether relationship duration moderates these effects, the SPSS PROCESS procedure (model 1) and 5000 bootstrap samples were adopted^61^. The interaction, women's Factor 1 X relationship duration, for men's behavior was significant (*B* = *0.0244, p* < *0.05, 95%CI* = *0.0024–0.0*465). The highest positive effect of women's on men's Factor 1 was relationship duration (+ 1 *SD*, 17 years) (*B* = *0.6439, p* < *0.001, 95%CI* = *0.3989–0.8*890) (Fig. 2).

**Figure 3:**
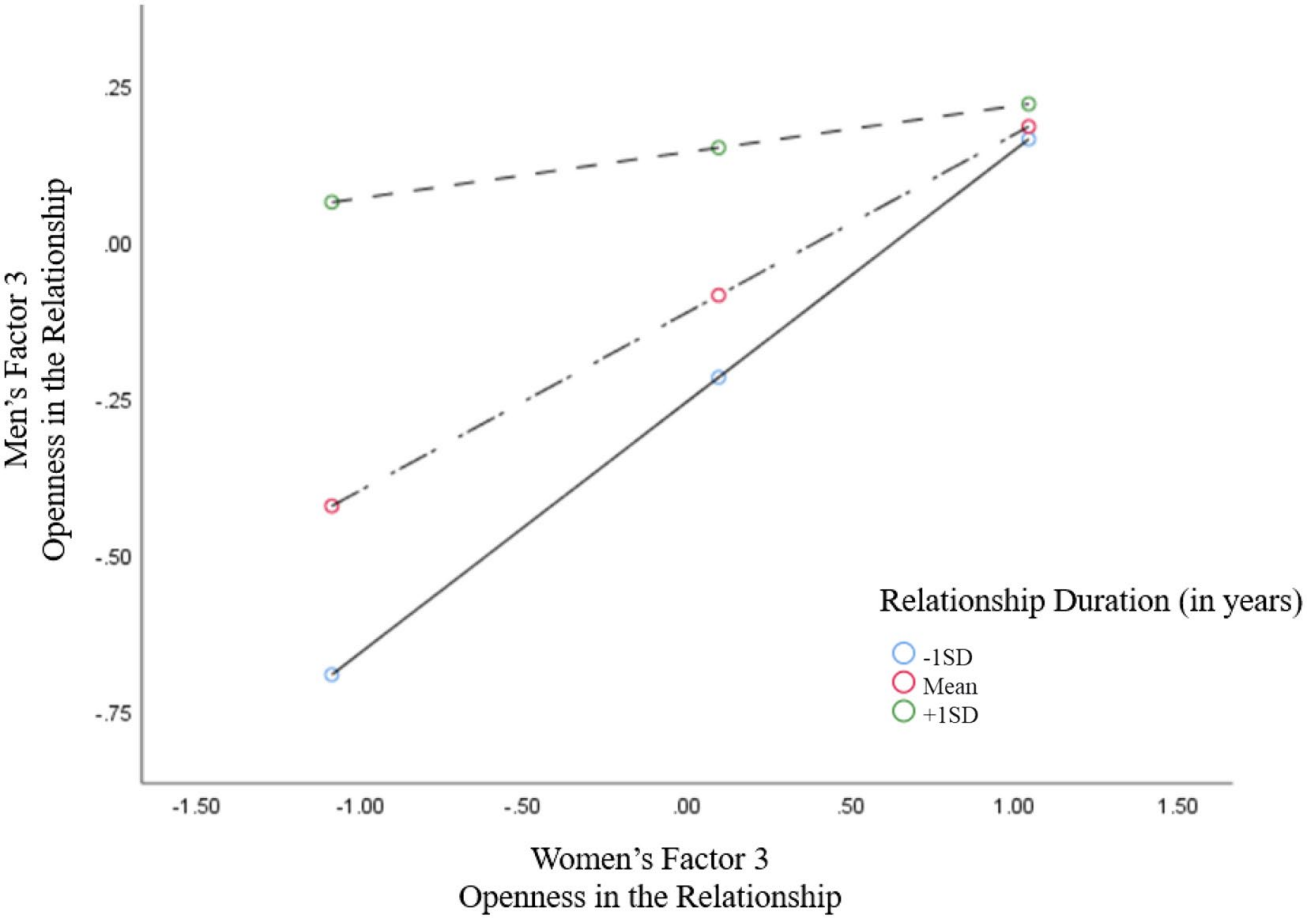
The results of the SPSS PROCESS procedure are presented. The interaction between the women's factor 3 and the relationship duration was significant in predicting the behavior of men. Conditional effects (simple slopes) of women's factor 3 (Openness in Relationship behaviors) on men's factor 3 behaviors at various values of relationship duration (plus (+ 1 SD) and minus (-1 SD) one standard deviation from the mean).

Furthermore, the interaction, women's Factor 3 X relationship duration, for men's behavior was also significant (*B* = *− 0.0234, p* < *0.05, 95%CI* = *− 0.0467 to − 0.0*001). When relationship duration was low (*− *1 *SD;* 3 years) and at the mean level (8 years) there were positive significant effects of women's openness behaviors on men's behaviors, respectively (*B* = *0.4019, p* < *0.001, 95%CI* = *0.1856–0.6*181), (*B* = *0.2847, p* < *0.001, 95%CI* = *0.1142–0.4*551). However, at high levels of relationship duration (+ 1 *SD*, 17 years) this effect was no longer significant (Fig. 3).

## Discussion

The first purpose of the current investigation was to construct and validate a comprehensive scale that assessed relational behaviors reported by both partners in a long-term relationship. The study also examined whether there were gender differences on one's scale factors in the behavior of one's partner. In addition, relationship duration as a moderator was also included in the scale development.

Study 1 described the development of the RBI scale yielded a very good reliability coefficient (α = 0.92). Study 2 assessed and characterized the scale's psychometric properties, as well as tested for differences in scale factors between men and women. Here we also examined whether women's responses predict their partners' behaviors, as well as to what extent relationship duration moderates these relationships. The findings showed three distinct factors: social companionship and affective behaviors, fulfilling obligations and duties as a partner, and behaviors associated with openness within a relationship. The correlation between men and their female partners on SAI (Factor 1) was (*r* = 0.48*, p* < 0.001) and on OR (Factor 3) was (*r* = 0.30*, p* < 0.01). As for FOD (Factor 2) (behaviors that reflect the fulfillment of the relationship's obligations and responsibilities), the correlation between genders was not significant.

As regards gender differences, there were significant differences between men and women on the second factor which represents behaviors associated with fulfilling the duties and obligations of one's partner. The results of the study indicated that gender significantly predicted behaviors related to fulfilling obligations and duties such as accepting the partner’s requests and needs, adhering to joint agreements, and fulfilling household tasks. In contrast, neither the first factor, which involves behaviors associated with social companionship and affective behavior interactions, nor the third factor, which involves behaviors related to openness in a relationship, displayed any distinct gender differences.

The importance of affective behaviors among couples and their potential benefit to a marital relationship has already been shown in previous studies^62,63^. Engaging in affective behavior interactions, such as displaying warmth towards one's partner, offering compliments, and expressing love, is likely to result in increased levels of commitment and satisfaction within a relationship^25^. Relationships in which men and women partners are aligned in social companionship, affective behaviors, and openness will probably achieve a more positive outcome. These findings are consistent with previous studies which showed that when partners have greater similarities in their relationship values, goals, and expectations, it is likely to be associated with a greater sense of trust and responsiveness. In turn, this will have a positive effect on relationship quality and improved functioning^64^. Additionally, communicating openly with one's partner is associated with a better understanding of each other's needs, feelings, and wants. When both partners feel supported and understood by the other, it can lead to a deeper bond and greater satisfaction in the relationship^65,66^.

The gender differences in FOD (Factor 2) are consistent with those reported by Stafford et al.^46^ who showed a weak link between gender and sharing tasks behaviors that may be perceived as the responsibilities of the couple. The significant gender differences observed for the second factor is consistent with social role theory. This theory suggests that men and women develop certain stereotypes or beliefs about their gender roles based on societal observations. In some societies, women are often still seen in nurturing roles, both at work and at home, which includes fulfilling obligations and duties such as accepting the partner’s requests and needs, adhering to joint agreements, and fulfilling household tasks. The results of the study here, which found a significant difference between men and women in behaviors related to fulfilling obligations and duties, provide empirical support for these theoretical expectations. These findings provide valuable insights into the ways in which social role theory can help us understand gender differences in behavioral interactions within couples. As such, the study fills a significant gap in the literature, offering a nuanced understanding of how gender roles influence behavioral interactions within long-term romantic relationships. This research could have important implications for relationship counseling and gender equality initiatives.

The similarities between men's and women's SAI and OR (Factor 1 and Factor 3) may be explained, to a large extent, by interdependence theory, which encompasses aspects such as identity expansion when partners coming together and a shift in motivations from self-centered to relationship-oriented^67,68^. According to interdependence theory, as romantic partners progress in their relationship, they influence each other's mental self-representations, fostering a greater sense of interconnectedness in terms of thoughts, emotions, behaviors, and mutual support^69–71^. This cognitive interdependence enhances their understanding and caregiving abilities, thus enabling them to meet each other's needs and desires, ultimately resulting in mutual influence on both their relationship dynamics and personal well-being^72^.

The duration of a relationship was found to be a significant moderator as the association between SAI of men and women was stronger for couples who have been together for a long time. These findings are consistent with Carstensen et al.^73^ who examined couples in long-term marital relationships and reported that they expressed less negativity and more affection. Relationship duration also moderates the association between women's openness behaviors and their partners, with a short-term relationship duration (3–8 years) leading to a more positive effect while a long-term relationship duration (17 years) was not found to have an effect. This finding appears to be consistent with other investigations which demonstrated that the use of openness decreases with relational length^16,47^.

In this study, we developed a comprehensive measure of romantic behavior interactions that demonstrated high convergent validity and enhanced our understanding of couples’ experiences within a romantic relationship. The present study addressed a gap in the literature by highlighting the need for a new scale that considers a range of behaviors significantly associated with relationship satisfaction and commitment^25^. Our scale fills this gap by considering behavioral interactions such as the fulfillment of partner obligations and duties, acceptance of the partner’s requests and needs, adherence to joint agreements, and completion of household tasks. Another key behavior considered here was openness within the relationship, which involves sharing personal needs, concerns, feelings, and emotions with the partner. This contributes to a more holistic understanding of relationship dynamics.

The findings here may very well contribute to enhancing relationship satisfaction and commitment. Notably, our scale differentiates from previous measures of romantic behaviors by considering the behaviors exhibited by both women and men, with a focus on long-term relationships. We examined the differences in scale factors between men’s and women’s behaviors as reported by cohabiting couples. Given the inconsistencies in the literature regarding men’s and women’s behaviors in romantic relationships, a primary objective was to determine whether our scale factors could distinguish between partner behaviors. Marital counselors and therapists could potentially use this scale to gain insight into various types of couples’ relational behaviors that have been overlooked in previous studies. By applying such knowledge, therapists can guide couples towards more constructive behaviors and cognitions that may help in improving relationship quality.

Although the RBI scale was found to add to our understanding of couples' behaviors, this research has some limitations. First, the cross-sectional design limited our ability to discuss potential differences over time. A longitudinal follow-up may provide answers to some of these concerns. Second, the study examined only heterosexual cohabiting couples in a romantic relationship. Third, most of the participants were in their mid-thirties and highly educated which limits the ability to generalize the results.

We view this research as an important step in understanding couples' behaviors. The use of RBI has the potential to describe relational behavior interactions more accurately, particularly the gender similarities, differences, and the moderating role of relationship duration. One of the significant contributions of this study is the prediction of men's behaviors from the partners' responses. This study generates a more in-depth and richer understanding of a couple's interactions and functioning.

Several studies in this field have focused primarily on young adults in dating relationships and newlywed couples, with only a limited number examining long-term relationships. Additionally, prior research often concentrated on relationship expectations and neglected the crucial behavioral component. The present study seeks to address these limitations by including the behavioral dynamics of cohabiting couples engaged in long-term relationships from a dyadic standpoint.

The outcomes of the study also have practical implications. Therapists and counselors working with couples facing relational challenges can pinpoint specific behavioral interactions within each partner, shedding light on various relationship outcomes such as commitment levels and overall relationship satisfaction. Moreover, these findings can contribute to the development of programs tailored to foster healthy romantic relationships. Such programs may offer individuals an opportunity to enhance self-awareness and emotional synchronization with one's partner. By facilitating discussions on behavioral interactions, cohabiting couples gain the ability to make more informed and improved decisions about their relationship. Therapists and counselors can play a pivotal role in helping couples recognize their behavioral patterns and allow for better promotion of constructive behaviors.

### Supplementary Information

Supplementary Figures.

## Data Availability

The study was generated at the University and the data are available from the corresponding author (T.H) on request.
