# Supplementary material for: Development and validation of the relational behavior interactions scale for couples
Source: Sci Rep. 2024 Apr 6;14:8086. doi: 10.1038/s41598-024-58901-2 (PMC10998839; doi:10.1038/s41598-024-58901-2)
Supplement: Supplementary file 1 — Supplementary Figures. [file 41598_2024_58901_MOESM1_ESM.docx]

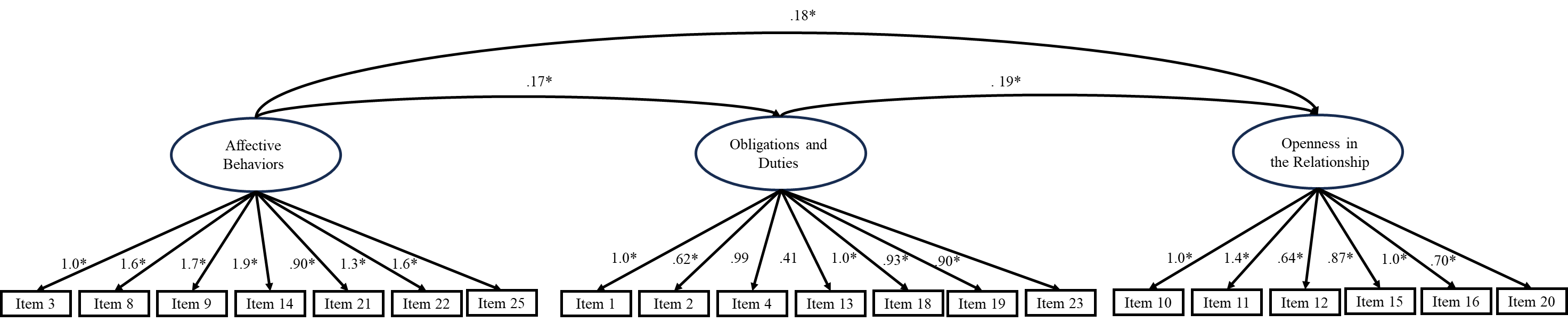


SI Figure 1. This figure represents the results from the CFA - Three factor model of Relational Behavior Interactions Scale and represents the model for women partners. Error variances and residues are omitted for clarity. Standardized corrected values are presented. All estimates are significant at p< .05. *p< .05, **p< .01


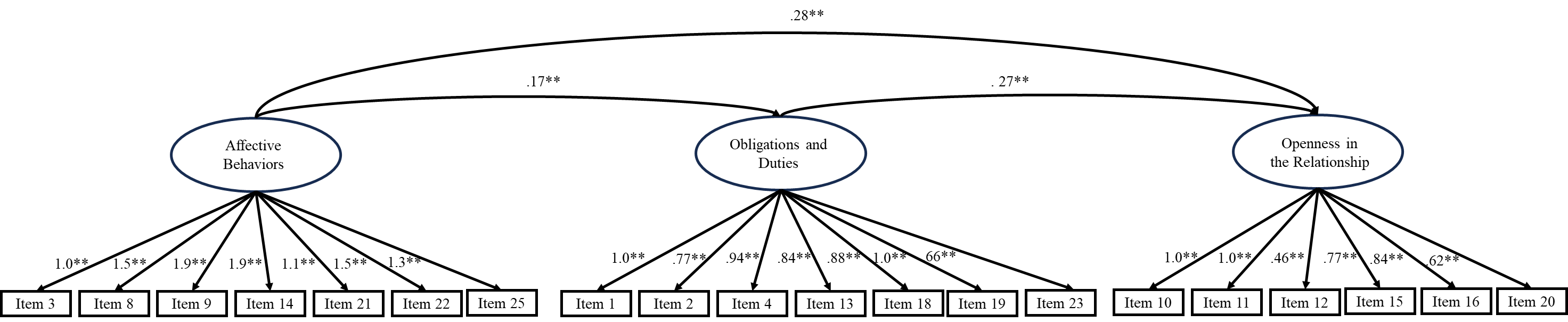


SI Figure 2. This figure represents the results from the CFA - Three factor model of Relational Behavior Interactions Scale and represents the model for men partners. Error variances and residues are omitted for clarity. Standardized corrected values are presented. All estimates are significant at p< .01. *p< .05, **p< .01
